# Significant individual variation in cardiac-cycle-linked cerebrospinal fluid production following subarachnoid hemorrhage

**DOI:** 10.1186/s12987-024-00587-9

**Published:** 2024-10-22

**Authors:** Per Kristian Eide, Ragnhild Marie Undseth, Øyvind Gjertsen, Lars Magnus Valnes, Geir Ringstad, Erika Kristina Lindstrøm

**Affiliations:** 1 Department of Neurosurgery, Oslo University Hospital-Rikhospitalet, Pb 4950, Nydalen, Oslo, N-0424 Norway; 2 Institute of Clinical Medicine, Faculty of Medicine, University of Oslo, Oslo, Norway; 3 KG Jebsen Centre for Brain Fluid Research, University of Oslo, Oslo, Norway; 4 The Interventional Centre, Oslo University Hospital-Rikhospitalet, Oslo, Norway; 5 Department of Mathematics, Faculty of Mathematics and Natural Sciences, University of Oslo, Oslo, Norway; 6 Department of Radiology, Oslo University Hospital- Rikshospitalet, Oslo, Norway; 7 Department of Geriatrics and Internal medicine, Sorlandet Hospital, Arendal, Norway; 8 Institute for Energy Technology, Kjeller, Norway

**Keywords:** Subarachnoid hemorrhage, Sylvian aqueduct, Cranio-cervical junction, Cerebrospinal fluid flow, Cerebrospinal fluid production

## Abstract

**Background:**

Spontaneous subarachnoid hemorrhage (SAH) often results in altered cerebrospinal fluid (CSF) flow and secondary hydrocephalus, yet the mechanisms behind these phenomena remain poorly understood. This study aimed to elucidate the impact of SAH on individual CSF flow patterns and their association with secondary hydrocephalus.

**Methods:**

In patients who had experienced SAH, changes in CSF flow were assessed using cardiac-gated phase-contrast magnetic resonance imaging (PC-MRI) at the Sylvian aqueduct and cranio-cervical junction (CCJ). Within these regions of interest, volumetric CSF flow was determined for every pixel and net CSF flow volume and direction calculated. The presence of acute or chronic hydrocephalus was deemed from ventriculomegaly and need of CSF diversion. For comparison, we included healthy subjects and patients examined for different CSF diseases.

**Results:**

Twenty-four SAH patients were enrolled, revealing a heterogeneous array of CSF flow alterations at the Sylvian aqueduct. The cardiac-cycle-linked CSF net flow in Sylvian aqueduct differed from the traditional figures of ventricular CSF production about 0.30–0.40 mL/min. In 15 out of 24 patients (62.5%), net CSF flow was retrograde from the fourth to the third and lateral ventricles, while it was upward at the cranio-cervical junction in 2 out of 2 patients (100%). The diverse CSF flow metrics did not distinguish between individuals with acute or chronic secondary hydrocephalus. In comparison, 4/4 healthy subjects showed antegrade net CSF flow in the Sylvian aqueduct and net upward CSF flow in CCJ. These net CSF flow measures also showed interindividual variability among other patients with CSF diseases.

**Conclusions:**

There is considerable inter-individual variation in net CSF flow rates following SAH. Net CSF flow in the Sylvian aqueduct differs markedly from the traditional ventricular CSF production rates of 0.30–0.40 mL/min in SAH patients, but less so in healthy subjects. Furthermore, the cardiac-cycle-linked net CSF flow rates in Sylvian aqueduct and CCJ suggest an important role of extra-ventricular CSF production.

**Supplementary Information:**

The online version contains supplementary material available at 10.1186/s12987-024-00587-9.

## Introduction

Spontaneous subarachnoid hemorrhage (SAH) typically arises from cerebral aneurysm rupture, bearing significant mortality and morbidity [1, 2]. Complications, such as cerebral vasospasms, delayed cerebral infarctions, and secondary hydrocephalus, contribute to outcomes. Disrupted cerebrospinal fluid (CSF) flow may play a crucial role for occurrence of complications post-SAH, such as secondary hydrocephalus, though the mechanistic underpinnings are not well understood [3–6]. Therefore, deeper comprehension of how SAH affects CSF flow and its link to secondary hydrocephalus is imperative.

One attractive hypothesis is that SAH induces inflammation and choroid plexus-driven CSF hypersecretion that combined with reduced CSF absorption at brain convexities results in hydrocephalus [7–11]. To explore this and other hypotheses, in vivo human studies assessing CSF disturbance are required. Currently, the most common in vivo examinations include measurements of CSF absorption capacity [12] or estimations of external CSF drainage volume [13], asl well as phase-contrast magnetic resonance imaging (PC-MRI) of CSF flow [14]. Whether acute or chronic secondary hydrocephalus necessitating CSF diversion is accompanied with specific CSF flow patterns remain undetermined.

This study employed cardiac-gated PC-MRI to evaluate cardiac-cycle-linked net CSF flow changes at the Sylvian aqueduct and cranio-cervical junction (CCJ) in patients with prior SAH. The observations in SAH patients were further compared with findings in healthy subjects and patients with carious CSF diseases.

## Methods

### Study cohort

The study cohort comprised SAH patients managed within the Department of Neurosurgery, Oslo University Hospital-Rikshospitalet, Norway, who were either hospitalized or had been hospitalized for their SAH. We included SAH patients with good grades who were able to undergo MRI. None of the study participants had a shunt or EVD at the time of MRI scanning. From our PC-MRI database, we retrieved healthy subjects and patients with CSF diseases who had undergone similar MRI acquisitions.

### Magnetic resonance imaging

We employed a 3 Tesla Philips Ingenia MRI scanner (Philips Medical Systems, Best, The Netherlands) for PC-MRI; the PC-MRI protocol was the same as previously reported [15, 16].

**Fig. 1:**
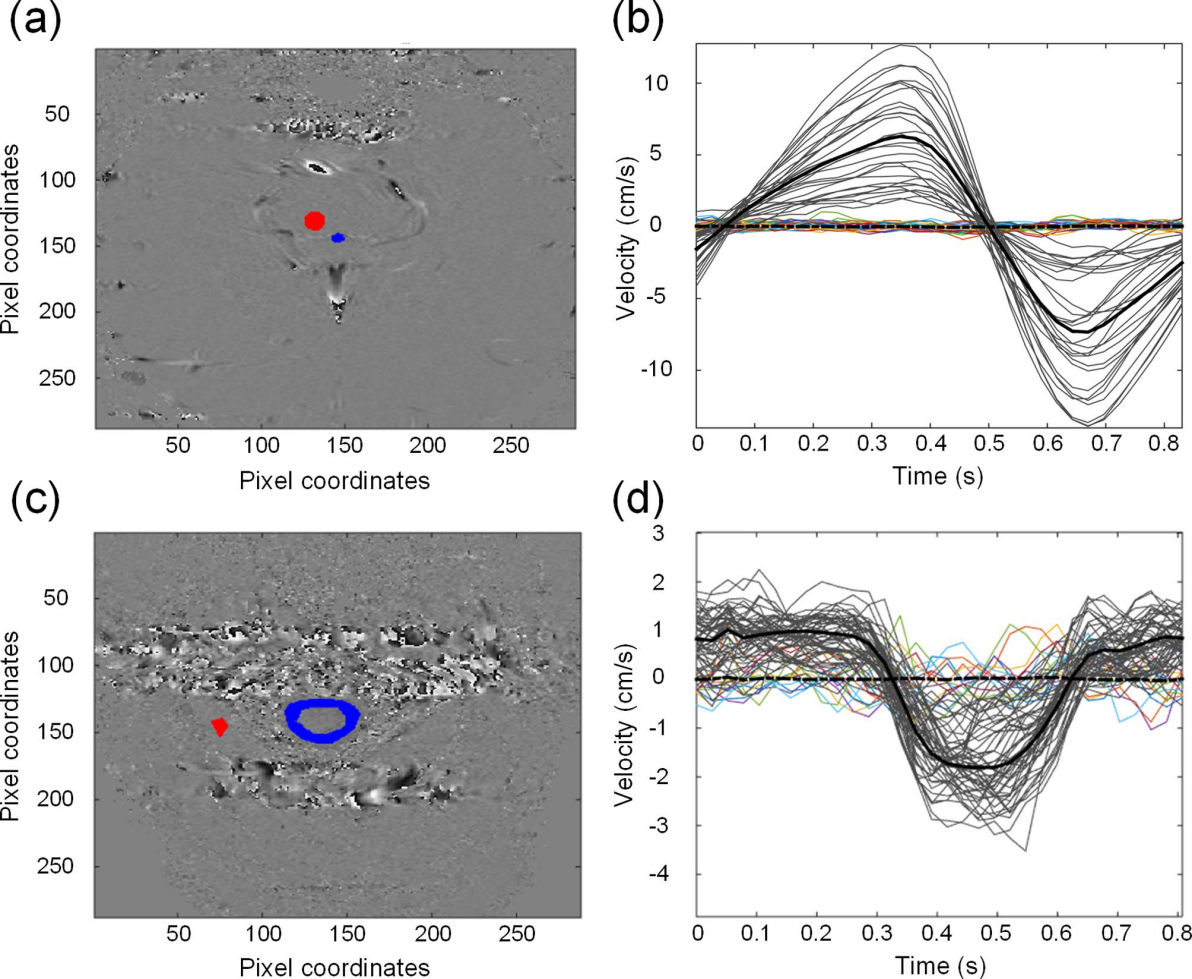
Cardiac-gated flow measurements from phase-contrast MRI of subject #24. (**a**) The region of interest (ROI) within the Sylvian aqueduct is shown in blue and reference ROIs in red. (**b**) Flow velocities within the Sylvian aqueduct were determined for every pixel within the ROI. Grey lines show the aqueduct pixel velocities with black line referring to mean velocity. The colored pixel velocities are from the reference ROI with black stippled line showing the mean velocity of the reference ROI. Additional examples from the SAH cohort of flow velocities within the Sylvian aqueduct are presented in Fig. 2. (**c**) The ROI within the CCJ is shown in blue and the reference ROI in red. (**d**) Flow velocities within the CCJ were determined for every pixel within the ROI. Grey lines show the CCJ pixel velocities with black line referring to mean velocity. The colored pixel velocities are from the reference ROI with black stippled line showing the mean velocity of the reference ROI

*Phase-contrast MRI utilized for assessing CSF flow.* The following regions of interest (ROIs) were defined: For the Sylvian aqueduct one ROI was placed within the aqueduct border and one in nearby brain tissue for reference velocities (Fig. 1a); flow velocities were determined at each pixel (Fig. 1b). For the cranio-cervical junction (CCJ), one ROI was placed within the CSF border of the CCJ and one in nearby brain tissue for reference velocities (Fig. 1c), and flow determined at each pixel (Fig. 1d). Raw data from PC-MRI were post-processed in MATLAB^®^ (MathWorks, Natick, United States); recorded velocities were linearly transformed from pixel values to centimeters per second using velocity encoding. We also determined reference velocities in a reference ROI, which served two purposes. First, it helps identify a low signal-to-noise ratio by comparing the signal with the reference ROI, which highlights the amount of noise present. Second, by calculating the mean velocity in the reference ROI, we can determine if there is any bias in the velocities. If the reference ROI mean velocity is not zero, this bias is subtracted from the velocities recorded in the CSF. Pixel velocities were adjusted for baseline shifts before further analysis. Aliased velocities, such as velocities exceeding the velocity encoding, were corrected using a filter. Mean velocity was obtained by averaging recorded velocities for each pixel within the ROI. We have provided a more detailed description of the methodology previously [15]. In brief, the volumetric flow rate (Q, ml/s) for each ROI was determined by summing pixel velocities over one cycle multiplied by pixel size:

$$\:Q\left(t\right)=\:\left(\sum\:_{i=1}^{n}{v}_{i\:}\right)\times\:dx\times\:dy$$

The stroke volume over one cycle was computed by discrete integration (trapezoidal method) of Q over time:

$$\:\text{Stroke\:volume=\:}\frac{\text{dt}}{\text{2}}\sum\:_{\text{i}\text{=1}}^{\text{n}}\text{(Q(}{\text{t}}_{\text{i+1}}\text{)+Q(}{\text{t}}_{\text{i}\text{\:}}\text{))}$$

CSF volumes in the Sylvian aqueduct, both cranially and caudally, were computed by integrating positive and negative volume fluxes over the course of one cycle. The CSF volumetric net flow rate during a single cycle (ml/cycle) was determined by summing the positive and negative CSF flux. Additionally, we calculated the net flow rate in mL/minute by multiplying the CSF net flow volume over one cardiac cycle by the heart rate (HR). Furthermore, we estimated the daily CSF volumetric net flow rate (L/24 hours) by multiplying the CSF net flow volume over one cardiac cycle by the heart rate (HR) and then by 1440 (minutes/day). The HR was determined over the MRI scan time, which was 6 min.

### Assessment of Hydrocephalus

#### Ventriculomegaly

Ventricular volume was estimated using FreeSurfer software (version 6.0; http://surfer.nmr.mgh.harvard.edu/) from T1 volume MRI scans. Additionally, a qualitative assessment of ventriculomegaly, indicative of secondary hydrocephalus, was conducted.

#### Acute hydrocephalus

Acute hydrocephalus was identified by the presence of radiological ventriculomegaly coupled with the need for CSF diversion via external ventricular drainage (EVD). At our institution, a low threshold exists for EVD placement in patients with acute SAH, primarily determined by reduced Glasgow Coma Scale (GCS) scores combined with enlarged cerebral ventricles.

#### Chronic hydrocephalus

Chronic hydrocephalus was diagnosed by qualitative assessment of ventriculomegaly, and by the necessity for shunt surgery.

#### Indication for shunt surgery

The decision for shunt surgery is guided by the clinical presentation along with computed tomography (CT) or MRI evidence of ventriculomegaly.

### Statistical analyses

The statistical analysis was performed using SPSS version 26 (IBM Corporation, Armonk, NY) and Stata/SE 16.1 (StataCrop LLC, College Station, TX). Statistical significance was accepted at the 0.05 level (two-tailed).

## Results

### Study cohort

Cardiac-gated phase-contrast (PC) MRI was conducted on 32 SAH patients; however, reliable flow velocities could not be obtained in eight patients due to signal noise, severe aliasing, or an unidentifiable aqueduct. The dataset consists of PC-MRI data from the Sylvian aqueduct in 24 patients and from the cranio-cervical junction (CCJ) in two patients. All included PC-MRI acquisitions were of good quality; no MRI acquisitions of acceptable quality were excluded.

Table 1 provides information about the study cohort of SAH patients. Patients were categorized based on the time since the bleed; the subgroups with SAH occurring < 3 months, 3–6 months, 6–12 months, or > 12 months before were comparable. No significant differences between subgroups were observed, except for a larger 4th ventricle volume in the 3–6 months subgroup compared to the > 6 months subgroups. Based on Glasgow Coma Scale (GCS) scores at admission and Glasgow Outcome Scale Extended (GOSE) scores at follow-up, these subjects were classified as good-grade SAH patients with favorable outcomes. Among them, a cerebral aneurysm was identified as the cause of SAH in 21 out of 24 patients [i.e., anterior communicating (ACOM) artery aneurysm in nine subjects, middle cerebral artery (MCA) aneurysm in six subjects, posterior communicating (PCOM) artery aneurysm in two subjects, anterior choroidal artery aneurysm in two subjects, superior cerebellar artery aneurysm in one and posterior inferior cerebellar artery in one subject]. In three cases, no cerebral artery aneurysm or vascular malformation was identified.

While 13/24 (54%) presented with acute hydrocephalus requiring external ventricular drainage, moderate radiological hydrocephalus at the time of MRI was observed in 7/24 (29%) patients (Table 1). Merely 1/24 (4%) individuals required permanent shunt.

**Table 1:** Demographic and clinical information about the subarachnoid haemorrhage (SAH) patients

	Subgroups separated by time from ictus					
	< 3 months	3–6 months	6–12 months	> 12 months	Significance	Total cohort
***Demographic***						
N	4	8	4	8		24
Sex (Female/Male; n, %)	2/2	4/4	3/1	6/2	ns	15/9
Age (years; average; stdev)	65.0 ± 5.7	60.1 ± 16.4	54.8 ± 2.5	61.1 ± 6.4	ns	60.4 ± 10.4
BMI (kg/m^2^; average; stdev)	28.0 ± 2.1	28.3 ± 7.3	23.5 ± 4.5	28.1 ± 4.7	ns	27.3 ± 5.5
***Acute stage***						
GCS at admission (median; ranges)	15 (15–15)	15 (13–15)	15 (14–15)	15 (15–15)	ns	15 (13–15)
Modified Fisher grade (median; ranges)	3 (3–4)	4 (1–4)	3 (1–3)	3 (1–4)	ns	3 (1–4)
Identified aneurysmal cause of bleed (n; %)	2/4 (50%)	7/8 (88%)	4/4 (100%)	8/8 (100%)	ns	21/24 (88%)
Surgical vs. EV aneurysmal repair (n; %)	1/2 (50%)	5/7 (71%)	4/4 (100%)	5/8 (63%)	ns	15/21 (71%)
Acute hydrocephalus (n; %)	2/4 (50%)	6/8 (75%)	3/4 (75%)	2/8 (25%)	ns	13/24 (54%)
***Follow-up***						
GOSE at follow-up (median; ranges)	7 (7–8)	7 (6–8)	7 (5–8)	5 (5–7)	ns	7 (5–8)
Qualitative moderate hydrocephalus (n; %)	2/4 (50%)	1/8 (13%)	1/4 (25%)	3/8 (38%)	ns	7/24 (29%)
***Volume of ventricles at follow-up***						
4th ventricle (mL)	1.8 ± 0.5	2.2 ± 0.4	1.4 ± 0.3	1.4 ± 0.6	^*^P < 0.05	1.8 ± 0.6
3rd ventricles (mL)	1.9 ± 0.5	1.9 ± 0.8	2.0 ± 0.6	1.6 ± 0.7	ns	1.8 ± 0.7
Lateral ventricles (mL)	37 ± 23	39 ± 19	39 ± 22	33 ± 24	ns	37 ± 20

BMI: Body mass index; EV: Endovascular; GCS: Glasgow Coma Score; GOSE: GOSE: Glasgow Outcome Score. Stdev: Standard deviation. Categorical data are presented as numbers with percentages, and continuous data as mean *±* standard deviation or median with ranges. Significant differences between groups were determined by analysis of variance (ANOVA) with Bonferroni corrected post-hoc tests to adjust for multiple comparisons, and differences between categorical data were determined by Pearson Chi-square test. Ns: Non-significant. ^*^P < 0.05: Significantly larger 4th ventricle in sub-group 3–6 months compared with subgroups 6–12 months and > 12 months

We also included all our healthy subjects (three men and one woman) aged 30.8 ± 8.5 years who had undergone the same PC-MRI acquisition with the same MRI scanner and identical protocol. These have been reported before, though we have not reported net flow rates (mL/min) before [15].

With regard to reproducibility of the present PC-MRI methodology, the four healthy subjects underwent the same PC-MRI acquisitions at five different time points, which disclosed high degree of reproducibility. For example, the average ± standard deviation of net flow rate pr cycle within the Sylvian aqueduct for five measurements in the four healthy subjects were: (ID 1) -0.002 ± 0.002 mL/cycle, (ID 2) -0.003 ± 0.002 mL/cycle, (ID 3) -0.002 ± 0.005 mL/cycle, and (ID 4) -0.003 ± 0.001 mL/cycle. The average ± standard deviation of net flow rate pr cycle within the CCJ for five measurements were: (ID 1) 0.033 ± 0.060 mL/cycle, (ID 2) 0.014 ± 0.028 mL/cycle, (ID 3) 0.112 ± 0.111 mL/cycle, and (ID 4) 0.164 ± 0.054 mL/cycle.

### Net CSF flow changes after SAH compared with healthy subjects

**Fig. 2:**
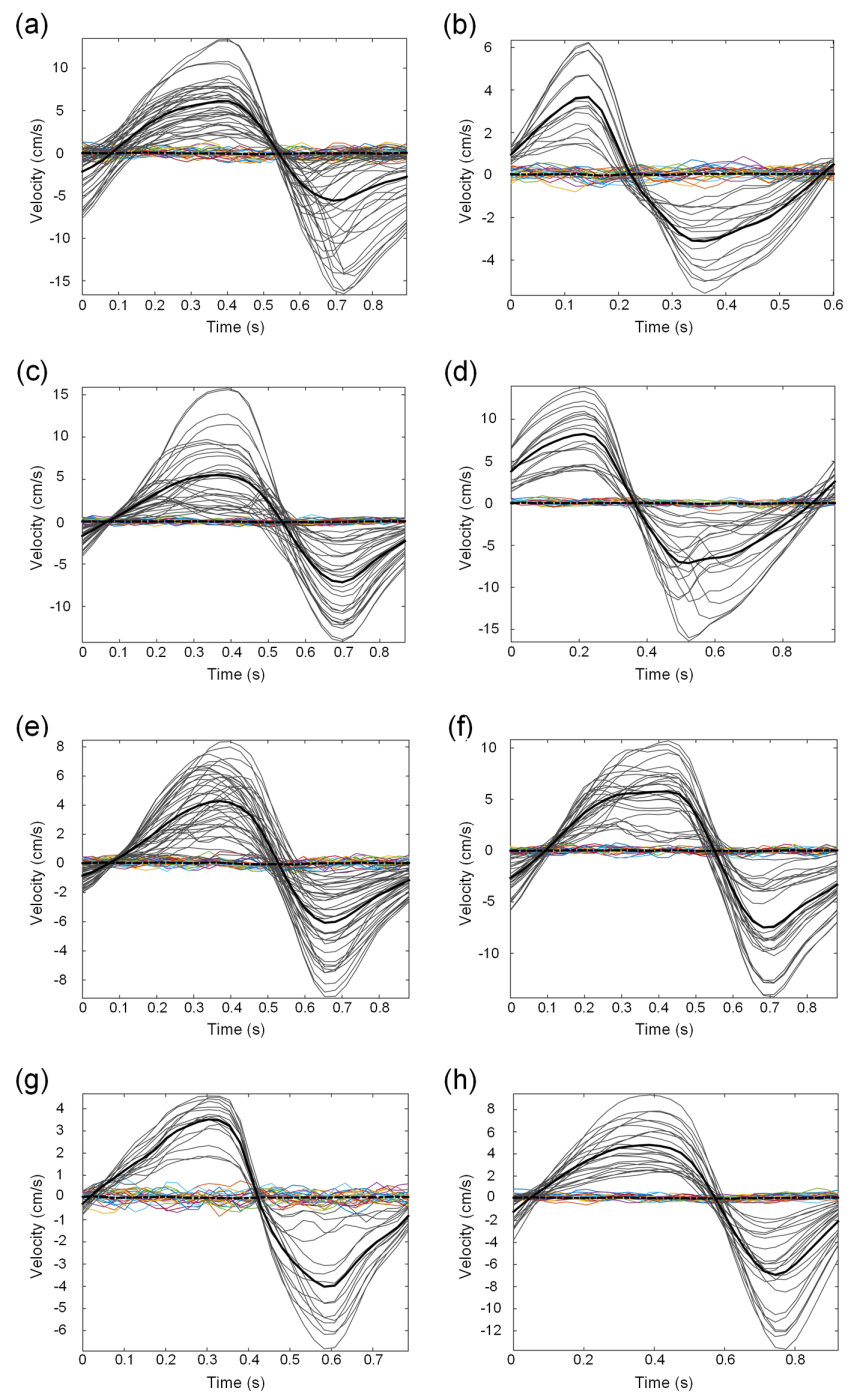
Cardiac-gated flow measurements within Sylvian aqueduct of SAH patients. Flow velocities within the Sylvian aqueduct were determined for every pixel within the region of interest (ROI). Grey lines show the aqueduct pixel velocities with black line referring to mean velocity. The colored pixel velocities are from the reference ROI with black stippled line showing the mean velocity of the reference ROI. For illustration, CSF flow velocities are shown for patients no. 1 (**a**) and 11 (**b**) with SAH < 3 months before, patients no. 4 (**c**) and 15 (**d**) with SAH 3–6 months earlier, patients no. 5 (**e**) and 16 (**f**) with SAH 6–12 months earlier, and patients no. 20 (**g**) and 23 (**h**) with SAH > 12 months before. The individual CSF flow estimates are further detailed in Supplementary Table 1

**Fig. 3:**
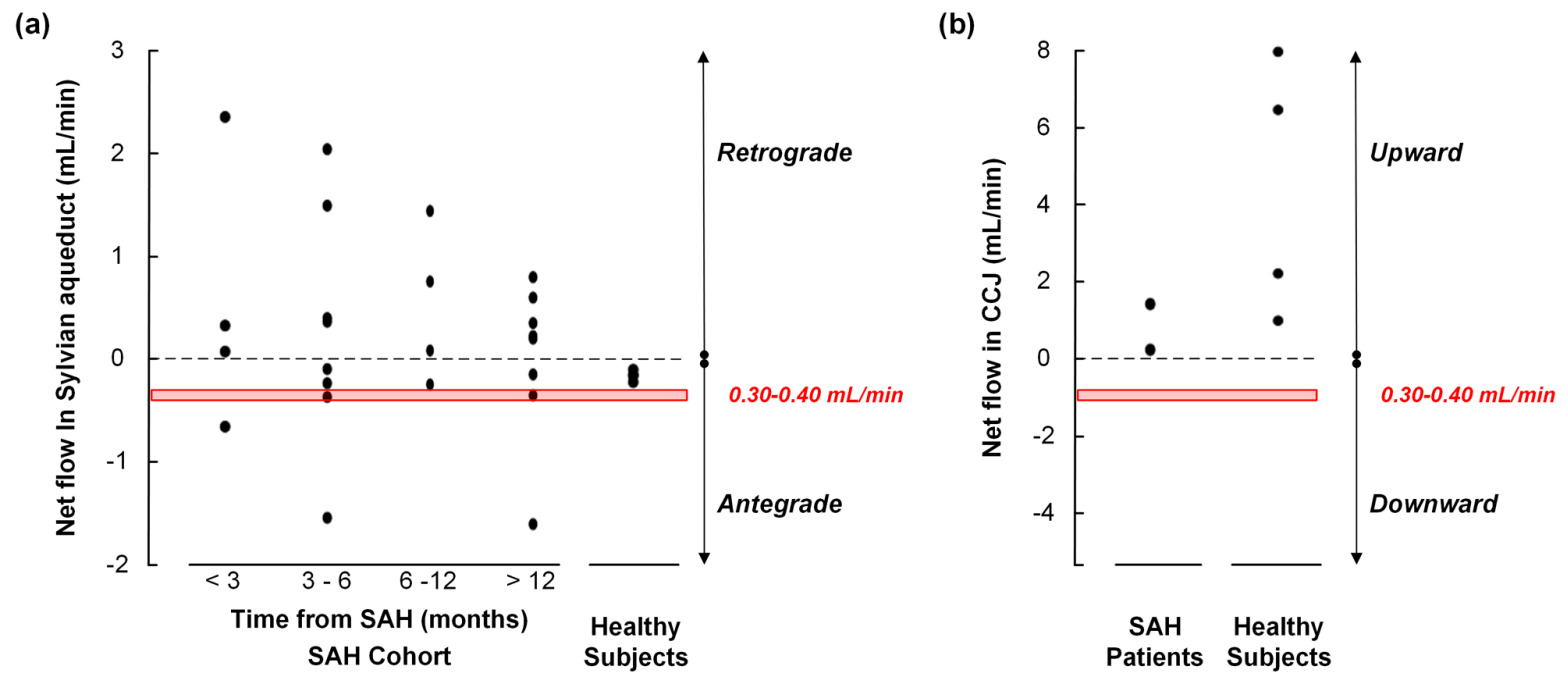
Cardiac-cycle-linked net CSF flow (mL/min) in (a) Sylvian aqueduct and (b) cranio-cervical junction. (**a**) The individual net CSF flow rates in Sylvian aqueduct of patients who had experienced an SAH either < 3 months (*n* = 4), 3–6 months (*n* = 8), 6–12 months (*n* = 4) or > 12 months (*n* = 8) before, and in four healthy subjects. Negative net flow values refer to antegrade net flow from 3rd to 4th cerebral ventricles, which we consider indicative of ventricular CSF production. Positive net flow values refer to retrograde flow from 4th to 3rd cerebral ventricles, suggesting extra-ventricular CSF production. The red box refers to the traditional concept of ventricular CSF production of 0.30–0.40 mL/min. Analysis of variance (ANOVA) with Bonferroni corrected post-hoc tests showed no differences between the groups (*P* = 0.79). (**b**) The individual net CSF flow rates in cranio-cervical junction of two SAH patients and four healthy subjects. Upward net CSF flow rates were seen in all six individuals and refer to net flow from the thecal sac to the intracranial compartment, which we consider indicative of CSF production within the thecal sac. The red box refers to the traditional concept of ventricular CSF production of 0.30–0.40 mL/min

Examples of CSF flow velocities within the Sylvian aqueduct of SAH patients within the various subgroups are presented in Fig. 2a-h. The individual cardiac-cycle-linked net CSF flow rates at the Sylvian aqueduct in 24 cases are presented in Supplementary Tables 1, and at the CCJ for two SAH subjects in Supplementary Table 2. The cardiac-cycle-linked net CSF flow changes in the Sylvian aqueduct are shown in Table 2. There were no statistical differences in net CSF flow estimates based on the time since the SAH. As depicted in Table 2, only 2 out of 24 patients exhibited antegrade net CSF flow volume rates between 0.3 and 0.4 mL/min. Retrograde net CSF flow from the 4th to the 3rd ventricle was observed in 15 out of 24 (62.5%) patients, while antegrade flow from the 3rd to the 4th ventricle was noted in 9 out of 24 (37.5%) patients. In comparison, 4/4 healthy subjects showed antegrade net CSF flow in the Sylvian aqueduct (*P* = 0.02, Pearson Chi-square test). The net CSF flow volumes did, however, not differ between SAH and healthy subjects (Table 2). Figure 3a illustrates the considerable variability in net CSF flow volumes among SAH patients, with 4/24 (17%) demonstrating retrograde net CSF flow > 1 mL/min and 2/24 (8%) showing antegrade net CSF flow > 1 mL/min.

**Table 2:** Cardiac-cycle-linked net CSF flow at Sylvian aqueduct and cranio-cervical-junction in SAH and healthy subjects

	SAH subgroups with variable time from ictus					SAH cohort	Healthy subjects	
	< 3 months	3–6 months	6–12 months	> 12 months	Significance			Significance
***Sylvian aqueduct***								
***Subgroup with antegrade net CSF flow***								
N	1 (25%)	4 (50%)	1 (25%)	3 (37.5%)		9/24 (37.5%)	4/4 (100%)	*P* = 0.02
Volume (mL/min)	0.64	0.55 ± 0.65	0.26	0.69 ± 0.77	ns	0.57 ± 0.57	0.16 ± 0.05	ns
***Subgroup with retrograde net CSF flow***								
N	3 (75%)	4 (50%)	3 (75%)	5 (62.5%)		15/24 (62.5%)	0	
Volume (mL/min)	0.92 ± 1.24	1.06 ± 0.82	0.72 ± 0.66	0.42 ± 0.25	ns	0.75 ± 0.72	-	
***Cranio-cervical junction***								
***Subgroup with upward net CSF flow***								
N	1	1				2/2 (100%)	4/4 (100%)	
Volume (mL/min)	1.429	0.241				0.84 ± 0.84	4.43 ± 3.39	ns

Significant differences between groups were determined by analysis of variance (ANOVA) with Bonferroni corrected post-hoc tests to adjust for multiple comparisons, and by independent samples t-test when SAH and Healthy subjects were compared. Ns: Non-significant

Net CSF flow at the CCJ was upward from thecal sac towards intracranial compartment in 2/2 SAH patients and 4/4 healthy subjects (Table 2). As shown in Fig. 3b, the volumes of upward net CSF flow in CCJ differed substantially.

**Fig. 4:**
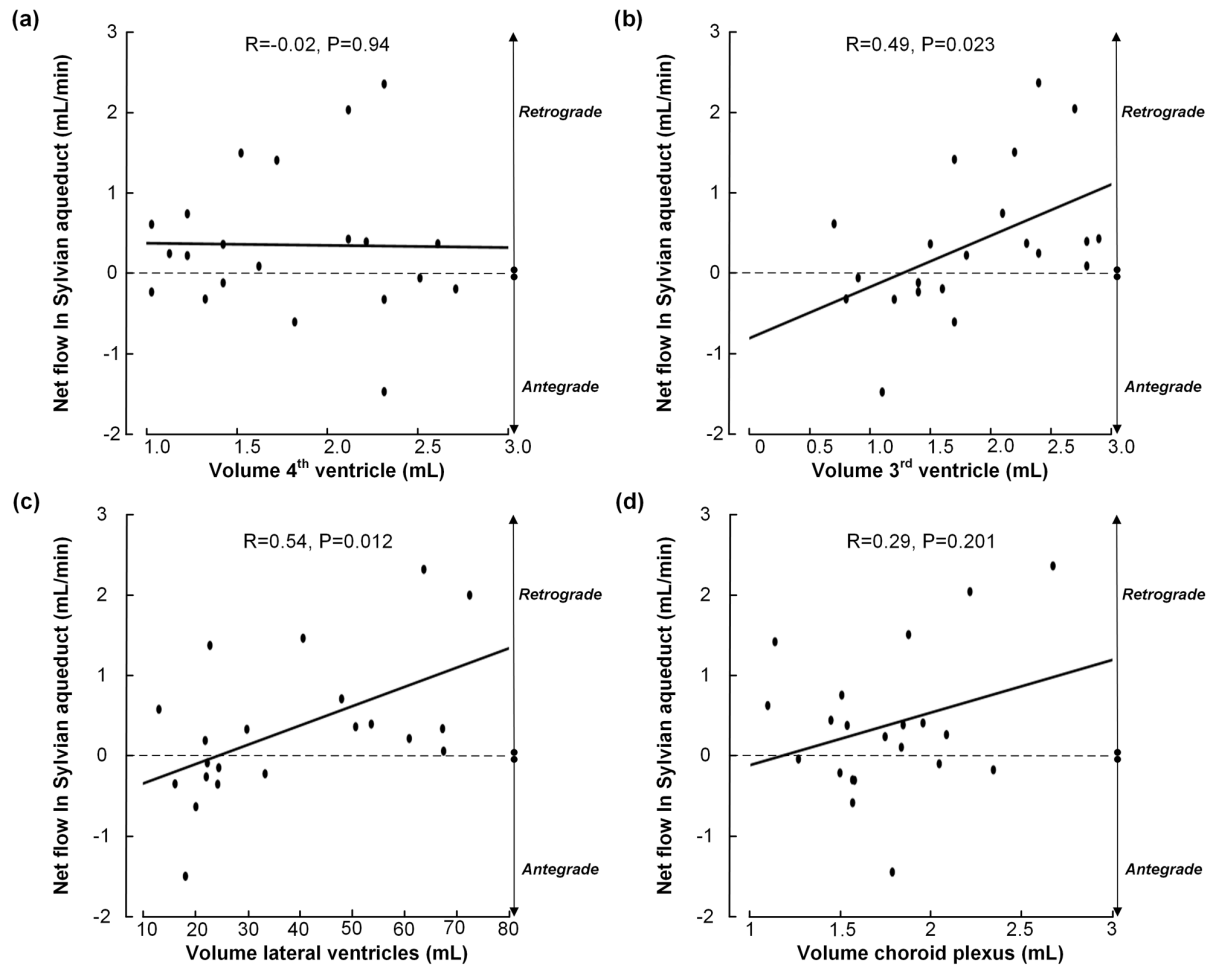
Correlation between CSF production (mL/min), estimated from Sylvian aqueduct net volumetric flow during the cardiac cycle, and volumes of ventricular size and choroid plexus. While net volumetric flow in the Sylvian aqueduct did not correlate with volume of 4th ventricle (**a**), there was a significant positive correlation between volume of 3rd ventricle and net flow (**b**) and the lateral ventricles (**c**). There was no significant correlation between net flow in Sylvian aqueduct and volume of choroid plexus (**d**). The fit line and Pearson correlation coefficient are shown. Negative net flow values refer to antegrade net flow from 3rd to 4th ventricle, and positive net flow values refer to retrograde flow from 4th to 3rd ventricle

Analysis of ventricular size showed no association between net CSF flow at the Sylvian aqueduct and the volume of the 4th ventricle (Fig. 4a). Nonetheless, a significant positive correlation was found between net CSF flow and the volumes of the 3rd (Fig. 4b) and lateral ventricles (Fig. 4c), indicating that increasing retrograde net CSF flow from the 3rd to the lateral ventricles was associated with increased volumes of these ventricles. There was no correlation between net CSF flow and volume of choroid plexus (Fig. 4d).

### Net CSF flow changes in patients with other CSF disorders

**Fig. 5:**
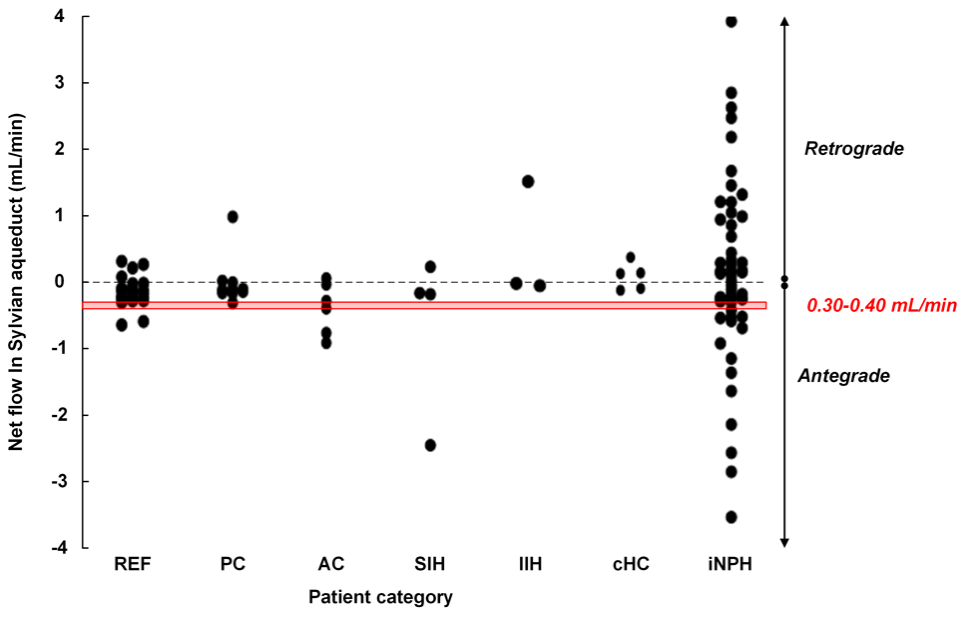
Cardiac-cycle-linked net CSF flow (mL/min) in Sylvian aqueduct of different patient categories. The net flow rates were retrieved from our PC-MRI database where PC-MRI acquisitions were done similarly as presented here. Other PC-MRI variables from these patients were previously reported by Eide et al. [16] and Lindstrøm et al. [15]. Patient categories: REF (Reference subjects with no diagnosed CSF disturbance), iNPH (idiopathic normal pressure hydrocephalus), cHC (communicating hydrocephalus other than iNPH), SIH (spontaneous intracranial hypotension), IIH (idiopathic intracranial hypertension), AC (symptomatic arachnoid cyst), PC (symptomatic non-hydrocephalic pineal cyst). The red box refers to the traditional concept of ventricular CSF production of 0.30–0.40 mL/min

**Fig. 6:**
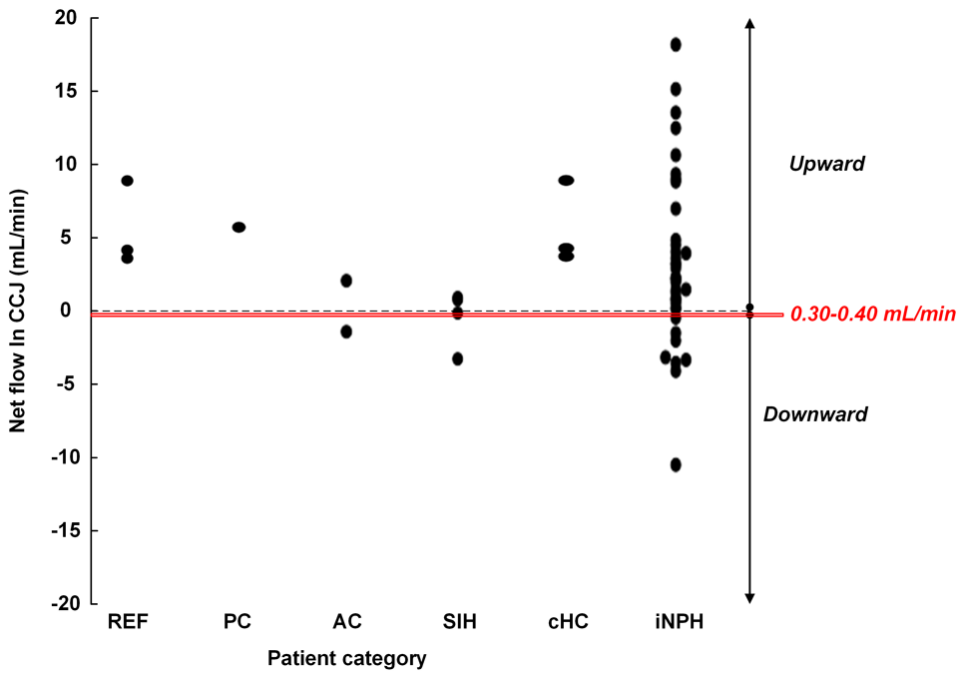
Cardiac-cycle-linked net CSF flow (mL/min) in cranio-cervical junction of different patient categories. The net flow rates were retrieved from our PC-MRI database where PC-MRI acquisitions were done similarly as presented here. Other PC-MRI variables from these patients were previously reported by Eide et al. [16] and Lindstrøm et al. [15]. Patient categories: REF (Reference subjects with no diagnosed CSF disturbance), iNPH (idiopathic normal pressure hydrocephalus), cHC (communicating hydrocephalus other than iNPH), SIH (spontaneous intracranial hypotension), AC (symptomatic arachnoid cyst), and PC (symptomatic non-hydrocephalic pineal cyst). The red box refers to the traditional concept of ventricular CSF production of 0.30–0.40 mL/minNet aqueduct CSF flow changes and secondary hydrocephalus after SAH

We further retrieved net CSF flow data from other patients with various CSF disorders. These patients have been reported on before [15, 16], but we have not previously presented net CSF flow rates in mL/min. Table 3 shows the net antegrade/retrograde CSF flow rates in Sylvian aqueduct for different CSF disease categories, as well as reference (REF) subjects with no identified CSF or neurological disorder. These figures further demonstrate significant variability in cardiac-cycle-linked CSF flow in various diseases; the variability is further illustrated in Fig. 5. Furthermore, Table 4 shows the variability in upward/downward net CSF flow at the CCJ, and Fig. 6 illustrates that upward net CSF flow at CCJ is common.

**Table 3:** Cardiac-cycle-linked CSF flow (mL/min) at Sylvian aqueduct for various diseases using the present PC-MRI methodology

	***Patient categories***							
Patient information	REF	iNPH	cHC	SIH	IIH	AC	PC	iNPH
N	23	32	5	4	3	7	11	21
Age	37 (19–64)	71 (46–80)	34 (24–54)	42 (25–56)	29 (28–53)	45 (22–62)	38 (19–47)	74 (56–84)
***Antegrade net CSF flow***								
N (%)	19 (83%)	18 (56%)	2 (40%)	3 (75%)	2 (67%)	5 (71%)	10 (91%)	7 (33%)
Volume (mL/min)	0.28 (0.08–0.69)	0.43 (0.01–3.46)	0.15 (0.14–0.16)	0.21 (0.20–2.43)	0.05 (0.03–0.06)	0.39 (0.04–0.89)	0.17 (0.03–0.35)	0.19 (0.07–2.78)
***Retrograde net CSF flow***								
N (%)	4 (17%)	14 (44%)	3 (60%)	1 (25%)	1 (33%)	2 (29%)	1 (9%)	14 (67%)
Volume (mL/min)	0.17 (0.10–0.24)	0.39 (0.12–2.30)	0.09 (0.08–0.32)	0.19	1.47	2.71 (0.05–5.38)	0.92	1.23 (0.05–4.05)
***Reference***	Eide et al. 2020 [16]							Lindstrøm et al. 2018 [15]

Net CSF flow in Sylvian aqueduct (mL/min) was retrieved from our PC-MRI database where PC-MRI acquisitions were done similarly as presented here. Other PC-MRI variables from these patients were previously reported by Eide et al. [16] and Lindstrøm et al. [15]. Data are presented as median values with ranges in parenthesis. Patient categories: REF (Reference subjects with no diagnosed CSF disturbance), iNPH (idiopathic normal pressure hydrocephalus), cHC (communicating hydrocephalus other than iNPH), SIH (spontaneous intracranial hypotension), IIH (idiopathic intracranial hypertension), AC (symptomatic arachnoid cyst), PC (symptomatic non-hydrocephalic pineal cyst). We consider antegrade net CSF flow as a measure of ventricular CSF production, and retrograde net CSF flow a measure of extra-ventricular CSF production

**Table 4:** Cardiac-cycle-linked CSF flow (mL/min) at cranio-cervical junction for various diseases using the present PC-MRI methodology

	Patient categories						
***Patient information***	REF	iNPH	cHC	SIH	IIH	AC	iNPH
N	4	18	3	4	1	2	22
Age	23 (19–47)	72 (46–80)	34 (32–54)	51 (25–69)	42	60 (58–62)	71 (45–84)
***Downward net CSF flow***							
N (%)	0	4 (22%)	0	2 (50%)	0	1 (50%)	6 (27%)
Volume (mL/min)		3.00 (0.06–10.52)		1.91 (0.36–3.47)		1.61	2.80 (0.12–3.72)
***Upward net CSF flow***							
N (%)	4 100%)	14 (78%)	3 (100%)	2 (50%)	1 (100%)	1 (50%)	16 (73%)
Volume (mL/min)	2.37 (0.29–15.83)	1.87 (0.23–6.56)	4.28 (3.73–8.89)	0.61 (0.54–0.68)	5.64	1.81	6.42 (0.44–17.50)
***Reference***	Eide et al. 2020 [16]						Lindstrøm et al. 2018 [15]

Net CSF flow in cranio-cervical-junction (mL/min) was retrieved from our PC-MRI database where PC-MRI acquisitions were done similarly as presented here. Other PC-MRI variables from these patients were previously reported by Eide et al. [16] and Lindstrøm et al. [15]. Data are presented as median values with ranges in parenthesis. Patient categories: REF (Reference subjects with no diagnosed CSF disturbance), iNPH (idiopathic normal pressure hydrocephalus), cHC (communicating hydrocephalus other than iNPH), SIH (spontaneous intracranial hypotension), IIH (idiopathic intracranial hypertension), and AC (symptomatic arachnoid cyst). We consider upward net CSF flow as a measure of CSF production within the thecal sac, while downward net CSF flow a measure of CSF production within the cranial compartment

The net CSF flow measures did neither differ between the subjects with (*n* = 13) / without (*n* = 11) acute hydrocephalus, nor between the subjects with (*n* = 7) / without (*n* = 17) qualitative radiological evidence of moderate hydrocephalus at the time of MRI (data not shown). Additionally, neither the extent of bleeding (modified Fisher grade) nor the presence of intraventricular hemorrhage correlated with CSF flow variables (data not shown).

## Discussion

The primary observation of this study is the considerable inter-individual variation in cardiac-cycle-linked net CSF flow changes after SAH. Antegrade net CSF flow at the Sylvian aqueduct was detected in 9 out of 24 SAH patients, while in 4/4 healthy subjects. Upward net CSF flow at the CCJ was observed in both 2/2 SAH patients and 4/4 healthy subjects. Only two patients exhibited net antegrade CSF flow at the Sylvian aqueduct, ranging between 0.30 and 0.40 mL/min, and merely two patients presented with antegrade net CSF flow compatible with ventricular CSF hypersecretion. However, qualitative indices of acute or chronic hydrocephalus did not reveal discernible patterns in net CSF flow.

### Net CSF flow patterns in humans

Although cardiac-gated PC-MRI solely assesses CSF flow during the cardiac cycle, neglecting variations during respiratory and slow vasomotor cycles, it provides insight into flow direction and variability. The present findings of retrograde net CSF flow from the 4th to the 3rd and lateral ventricles in the majority of SAH patients compare with previous findings in patients with idiopathic normal pressure hydrocephalus (iNPH) who demonstrate reflux to ventricles of an intrathecal CSF tracer [15–18]. The presently demonstrated net upward CSF flow at the CCJ compares with observations of rapid passage to cisterna magna of a CSF tracer injected intrathecal at the lumbar levels [15, 16]. It is worth noting that the present observations refer to changes during the cardiac cycle; others previously reported upward CSF movement at upper thoracic level in response to forced inspiration in 11/12 healthy volunteers [19].

Notably, the present net CSF flow volumes do not refer to total CSF production, only to the CSF flow changes during the cardiac cycle, hence the term cardiac-cycle-linked net CSF flow. Various methods have been employed to assess CSF production, revealing considerable variability in CSF production rates [13, 20]. Some authors even argue against the existence of net CSF flow at the Sylvian aqueduct [21]. There is, however, an increasing awareness that the traditional figures of ventricular CSF production in the range 0.30 to 0.40 mL/min (approximately 430 to 580 mL/day) may not be correct [22, 23]. While it may have relevance for healthy subjects, as indicated here, the case may be very different in disease states. In the present cohort, these CSF production rates were observed in only 2/24 (8.3%) patients, only considering the cardiac component.

Traditionally the choroid plexus has been considered the primary source of CSF production, though emerging evidence suggests the existence of diverse sites for CSF production [23]. The lack of correlation between choroid plexus volume and CSF flow variables may indicate that choroid plexus volume not directly associates with CSF flow. Most likely, there is a significant extra-ventricular CSF production, which is supported by the present observations of retrograde net CSF flow at the Sylvian aqueduct and upward net CSF flow at the CCJ. The direction of net CSF flow as a measure of CSF production should theoretically be from higher to lower pressure, suggesting movement away from the production site towards the absorption site.

### CSF disturbance following SAH

It is well established that spontaneous SAH significantly disrupts CSF homeostasis, often leading to secondary hydrocephalus characterized by radiological ventriculomegaly or the clinical necessity for CSF diversion [3]. Previous studies on CSF disturbance after SAH have primarily focused on secondary hydrocephalus post-SAH; approximately one-third of SAH patients may require a shunt post-SAH though the proportion varies extensively between studies as do the identified risk factors [24–26].

The hypothesis that SAH induces inflammation and choroid plexus-driven CSF hypersecretion [7–11] was only partly supported by the present observations. Merely 2/24 (8%) patients demonstrated increased antegrade CSF flow via the Sylvian aqueduct during the cardiac cycle (1.577 mL/min and 1.513 mL/min), which could be indicative of ventricular hypersecretion of CSF. The retrograde net CSF flow at the Sylvian aqueduct does not align with ventricular CSF hypersecretion. In addition, there was no correlation between choroid plexus volume and net CSF flow at Sylvian aqueduct.

In this study, the net CSF flow changes were neither associated with the presence of acute hydrocephalus, nor by ventriculomegaly at the time of MRI. Furthermore, despite the large variability in net CSF flow changes, both at the Sylvian aqueduct and the CCJ, only one of 24 patients required a permanent shunt. This discrepancy highlights the complexity of factors influencing the need for shunt placement, suggesting that neither CSF flow alterations nor ventricular size alone can reliably predict shunt requirement. The pathophysiological mechanisms underlying shunt dependency post-SAH remain incompletely understood, warranting further investigation.

Here, we focus on secondary hydrocephalus, but it should be mentioned that CSF disturbances may perhaps also contribute to other post-SAH complications, such as fatigue and cognitive decline. Further studies are required to address these questions.

### Limitations

Estimation of total CSF production remains challenging due to its variations over the cardiac, respiratory, and slow vasomotor cycles, and not least methodological limitations [20]. Currently, we are left with approaches only providing part of the picture. In this regard, insights about CSF flow during the cardiac cycle provided by cardiac-gated PC-MRI may be useful.

Among the SAH patients, we had to exclude eight of 32 patients due to signal noise, severe aliasing, or an unidentifiable aqueduct. This rather high failure rate affecting measurements in eight subjects might be related to their SAH where the bleed may have impacted aqueduct morphology and function. Inclusion of poor-quality CSF flow acquisitions of these eight individuals was not done to avoid erroneous interpretations. The quality of PC-MRI acquisitions of the 24 included subjects were of good quality. Our methodology of determining flow velocities within the individual pixels of the Sylvian aqueduct and CCJ provides for a more detailed and accurate analysis. In addition, we determined the signal within the reference ROI to assess signal to noise ratio.

It remains, however, a limitation that the estimation of net CSF flow volumes from cardiac-gated PC-MRI do not provide any measure of the total CSF production. Therefore, we here refer to cardiac-cycle-linked CSF production. CSF flow volumes are dominated by respiration more than cardiac pulsations [27], and respiration, particularly deep breathing, affect the CSF flow estimated by cardiac gated PC-MRI [28–30]. which may also impact the CSF flow volumes during the cardiac cycle. However, despite its limitations, the cardiac-gated PC-MRI capture the inter-individual variation and the deviation from traditional CSF production rates. In addition, the presently used methodology provides increased accuracy of flow velocity measurements as the velocities are estimated for each pixel within the ROI; the average number of pixels for the Sylvian aqueduct was 26 (ranges 13–66).

Another limitation is that we included merely four healthy subjects who were younger than the SAH patients and also included a higher proportion of male sex. It has previously been shown the CSF flow variables of the Sylvian aqueduct correlate with age and sex [31]. The non-significant differences in net CSF flow between SAH and healthy subjects could thus be influenced by age and sex differences.

Finally, the present study cohort, comprising good-grade SAH patients with favorable outcomes, limiting the generalizability of findings to all SAH patients.

## Conclusions

Spontaneous SAH induces person-specific changes in CSF flow patterns, with large inter-individual variation in net CSF flow changes. Retrograde net CSF flow at the Sylvian aqueduct is prevalent, while increased antegrade net CSF flow at the Sylvian aqueduct, indicative of ventricular CSF hypersecretion, is rare. There is no direct association between net CSF flow changes and radiological ventriculomegaly or shunt dependency.

## Electronic supplementary material

Below is the link to the electronic supplementary material.

Supplementary Material 1

## Data Availability

No datasets were generated or analysed during the current study.

## Abbreviations

ACOM: Anterior communicating
CCJ: Cranio-cervical-junction
CSF: Cerebrospinal fluid
CT: Computed tomography
EVD: External ventricular drainage
GCS: Glashow Coma Score
GOSE: Glasgow Outcome Scale Extended
HR: Heart rate
iNPH: Idiopathic normal pressure hydrocephalus
MCA: Middle cerebral artery
MRI: Magnetic resonance imaging
PCOM: Posterior communicating
PC-MRI: Phase-contrast magnetic resonance imaging
ROI: Region of interest
SAH: Subarachnoid hemorrhage
