# Supplementary material for: Significant individual variation in cardiac-cycle-linked cerebrospinal fluid production following subarachnoid hemorrhage
Source: Fluids Barriers CNS. 2024 Oct 22;21:85. doi: 10.1186/s12987-024-00587-9 (PMC11495023; doi:10.1186/s12987-024-00587-9)
Supplement: Supplementary file 1 — Supplementary Material 1 [file 12987_2024_587_MOESM1_ESM.pdf]

Supplementary Material

Significant Individual Variation in Cardiac-Cycle-Linked Cerebrospinal Fluid Production Following Subarachnoid Hemorrhage

Per Kristian Eide, MD, PhD[1,2,3*], Ragnhild Marie Undseth, MD, PhD[4], Øyvind Gjertsen, MD,[6] Lars Magnus Valnes, PhD,[1,5], Geir Ringstad, MD, PhD,[2,3,6,7], Erika Kristina Lindstrøm, PhD[8]

[1]*Department of Neurosurgery, Oslo University Hospital – Rikshospitalet, Oslo, Norway.*
[2]*Institute of Clinical Medicine, Faculty of Medicine, University of Oslo, Oslo, Norway.*
[3]*KG Jebsen Centre for Brain Fluid Research, University of Oslo, Oslo, Norway*
[4]*The Interventional Centre, Oslo University Hospital – Rikshospitalet, Oslo, Norway*
[5]*Department of Mathematics, Faculty of Mathematics and Natural Sciences, University of Oslo, Norway.*
[6]*Department of Radiology, Oslo University Hospital- Rikshospitalet, Oslo, Norway.*
[7]*Department of Geriatrics and Internal medicine, Sorlandet Hospital, Arendal, Norway.*
[8]*Institute for Energy Technology, Kjeller, Norway*

Corresponding author:
Professor Per Kristian Eide, MD PhD
Department of Neurosurgery
Oslo University Hospital - Rikshospitalet
Pb 4950 Nydalen,
Phone: +47 91649419
Fax: +47-23074310
N-0424 Oslo, Norway
p.k.eide@medisin.uio.no

Supplementary Table 1. Cardiac-cycle-linked estimates of CSF flow at the Sylvian aqueduct in patients with subarachnoid hemorrhage (SAH).

PatID	Time from SAH	HR	Region of interest (ROI)		Calculations per cardiac cycle						Estimates	
			Number pixels	Area (cm^2)	dP (mmHg/cm)	Positive flow/cycle (mL)	Negative flow/cycle (mL)	Total flow/cycle (mL)	Net flow/cycle (mL)	Net direction of flow	Net flow (mL/min)	Net flow (L/24 h)
1	< 3mths	65	39	0.092	0.079	0.178	-0.142	0.319	0.036	R	2.336	3.472
2	3-6 mths	84	18	0.043	0.041	0.025	-0.029	0.053	-0.004	A	-0.357	-0.533
3	6-12 mths	78	13	0.031	0.065	0.032	-0.035	0.067	-0.003	A	-0.264	-0.393
4	3-6 mths	67	33	0.078	0.094	0.133	-0.127	0.261	0.006	R	0.397	0.591
5	6-12 mths	66	41	0.097	0.060	0.117	-0.096	0.213	0.021	R	1.383	2.056
6	< 3mths	79	34	0.080	0.115	0.132	-0.131	0.263	0.001	R	0.082	0.121
7	6-12 mths	55	19	0.045	0.075	0.095	-0.082	0.177	0.013	R	0.713	1.060
8	>12 mths	78	24	0.057	0.021	0.013	-0.033	0.046	-0.020	A	-1.577	-2.347
9	3-6 mths	62	18	0.043	0.032	0.035	-0.037	0.072	-0.001	A	-0.090	-0.133
10	3-6 mths	57	18	0.043	0.055	0.040	-0.067	0.107	-0.027	A	-1.513	-2.249
11	< 3mths	96	17	0.040	0.066	0.022	-0.029	0.051	-0.007	A	-0.639	-0.957
12	3-6 mths	70	29	0.069	0.094	0.120	-0.115	0.235	0.005	R	0.363	0.540
13	3-6 mths	71	34	0.080	0.061	0.115	-0.094	0.210	0.021	R	1.473	2.190
14	< 3mths	57	24	0.057	0.117	0.151	-0.146	0.297	0.006	R	0.332	0.493
15	3-6 mths	61	20	0.047	0.097	0.109	-0.113	0.221	-0.004	A	-0.226	-0.335
16	6-12 mths	66	28	0.066	0.096	0.124	-0.123	0.246	0.001	R	0.058	0.087
17	3-6 mths	60	66	0.156	0.055	0.243	-0.209	0.452	0.034	R	2.013	2.993
18	>12 mths	88	14	0.033	0.072	0.029	-0.033	0.062	-0.004	A	-0.352	-0.463
19	>12 mths	73	21	0.050	0.056	0.059	-0.049	0.108	0.011	R	0.777	1.155
20	>12 mths	74	15	0.035	0.066	0.031	-0.033	0.064	-0.002	A	-0.150	-0.223
21	>12 mths	68	36	0.085	0.051	0.086	-0.081	0.167	0.005	R	0.339	0.503
22	>12 mths	72	26	0.061	0.035	0.046	-0.042	0.088	0.003	R	0.216	0.374
23	>12 mths	63	22	0.052	0.081	0.088	-0.085	0.174	0.003	R	0.191	0.285
24	>12 mths	52	20	0.047	0.055	0.081	-0.070	0.150	0.011	R	0.582	0.866

HR: Heart rate average over the 6-minute MRI acquisition period. dP: Pressure gradient. Positive flow refers to flow in retrograde (upward) direction. Negative flow refers to flow in antegrade (downward) direction. R: Retrograde in Sylvian aqueduct. A: Antegrade in Sylvian aqueduct.

Supplementary Table 2. Cardiac-cycle-linked estimates of CSF flow at the cranio-cervical junctions in patients with subarachnoid hemorrhage (SAH).

PatID	Time from SAH	HR	Region of interest (ROI)		Calculations per cardiac cycle						Estimates	
			Number pixels	Area (cm^2)	dP (mmHg/cm)	Positive flow/cycle (mL)	Negative flow/cycle (mL)	Total flow/cycle (mL)	Net flow/cycle (mL)	Net direction of flow	Net flow (mL/min)	Net flow (L/24 h)
23	>12 mths	93	839	1.98	0.029	0.405	-0.390	0.796	0.015	U	1.429	2.137
24	>12 mths	72	570	1.35	0.040	0.551	-0.548	1.099	0.003	U	0.241	0.359

HR: Heart rate average over the 6-minute MRI acquisition period. dP: Pressure gradient. Positive flow refers to flow in retrograde (upward) direction. Negative flow refers to flow in antegrade (downward) direction. U: Upward in CCJ
